# Leukocyte telomere length and personality: associations with the Big Five and Type D personality traits

**DOI:** 10.1017/S0033291717002471

**Published:** 2017-09-11

**Authors:** D. Schoormans, J. E. Verhoeven, J. Denollet, L. van de Poll-Franse, B. W. J. H. Penninx

**Affiliations:** 1 Department of Medical and Clinical psychology, CoRPS – Center of Research on Psychology in Somatic diseases, Tilburg University, Tilburg, The Netherlands; 2 Department of Psychiatry VU University Medical Center, Amsterdam Public Health Research Institute, Amsterdam, The Netherlands; 3 Netherlands Comprehensive Cancer Organization (IKNL), Utrecht, The Netherlands; 4 Department of Psychosocial Research and Epidemiology, The Netherlands Cancer Institute, Amsterdam, The Netherlands

**Keywords:** Big Five, cellular aging, leukocyte telomere length, personality, Type D personality

## Abstract

**Backgrounds:**

Accelerated cellular ageing, which can be examined by telomere length (TL), may be an overarching mechanism underlying the association between personality and adverse health outcomes. This 6-year longitudinal study examined the relation between personality and leukocyte telomere length (LTL) across time among adults with a wide age-range.

**Methods:**

Data from the Netherlands Study of Depression and Anxiety were used and included patients with a depression and/or anxiety disorder and healthy controls. Overall, 2936 persons (18–65 years, 66% female) had data on LTL at baseline and 1883 persons had LTL at 6-year follow-up. The Big Five personality traits (neuroticism, extraversion, openness to experience, agreeableness, and conscientiousness) and Type D personality were assessed.

**Results:**

Neuroticism was negatively (*B* = −2.11, *p* = 0.03) and agreeableness was positively (*B* = 3.84, *p* = 0.03) related to LTL measured across two time points, which became just non-significant after adjusting for somatic health, lifestyle factors, and recent life stress (*B* = −1.99, *p* = 0.06; and *B* = 3.01, *p* = 0.10). Type D personality was negatively (*B* = −50.16, *p* < 0.01) related to LTL across two time points, which still remained statistically significant after full adjustment (*B* = −47.37, *p* = 0.01). Associations did not differ by age, gender, and current psychiatric status.

**Conclusions:**

The Big Five traits high neuroticism and low agreeableness, and Type D personality were associated with shorter LTL measured across a 6-year period. Associations with the Big Five traits became non-significant after controlling for somatic health, lifestyle factors, and recent life stress, yet similar trends were observed. Type D personality remained independently associated with shorter LTL after full adjustment.

## Introduction

Personality can be defined in various ways, but the most widely accepted personality framework is the Five Factor Model (John *et al.* 2008). This model consists of the following five personality traits: neuroticism (emotionally unstable and easily anxious), extraversion (outgoing), conscientiousness (efficient and organized), agreeableness (friendly and compassioned), and openness to experience (inventive and curious), the so-called ‘Big Five’. There is increasing evidence that aspects of personality are associated with adverse health outcomes. In detail, individuals who score low on conscientiousness have an increased risk of early mortality (Kern & Friedman, 2008; Jokela *et al.* 2013). Furthermore, high neuroticism (Wilson *et al.* 2004; Chapman *et al.* 2010) and low agreeableness (Weiss & Costa, 2005) have been related to an increased risk of mortality, although these findings were not confirmed by a meta-analysis (Jokela *et al.* 2013). Moreover, the Big Five personality traits high neuroticism and low conscientiousness have been related to adverse cardiac events among cardiac patients (Jokela *et al.* 2014; McCann, 2014).

More recently, the distressed (Type D) personality was introduced when studying the role of personality in a cardiovascular population (Denollet, 2005). Type D personality refers to the combination of the traits negative affectivity and social inhibition (Denollet, 2005). People scoring high on negative affectivity have a tendency to experience negative emotions, whereas individuals who score high on social inhibition have a tendency to inhibit self-expression. Persons with high scores on *both* personality traits are classified as having a Type D personality (Denollet, 2005). Although Type D personality is positively and negatively related to the Big Five traits neuroticism and extraversion respectively, it is a distinct construct (De Fruyt & Denollet, 2002). Studies show mixed findings, where some studies report that persons with a Type D personality have an increased risk of mortality (Denollet, 2013; Denollet *et al.* 2013), others did not (Pelle *et al.* 2010). Furthermore studies have linked Type D personality to poor outcomes in aging-related somatic illnesses, such as cardiovascular disease (CVD) and cancer (Denollet, 2005; Mols *et al.* 2012).

Although the mechanisms underlying the associations between personality traits and health are not fully understood, both behavioural and pathophysiological mechanisms may be involved. For example, higher levels of agreeableness have been related to lower levels of obesity (Sutin *et al.* 2011) and might thus be a protective factor, while higher levels of neuroticism, lower levels of agreeableness, and Type D personality have been associated with higher levels of inflammation (Conraads *et al.* 2006; Sutin *et al.* 2010). Furthermore, high neuroticism and Type D personality have been associated with poor health behaviours such as smoking (Lerman *et al.* 2000; Pedersen *et al.* 2004). Accelerated cellular ageing may be underlying the relation between these personality traits and poor health outcomes. According to the exposome paradigm of cellular aging, the above mentioned behavioural and pathophysiological factors are exposures possibly impacting cellular ageing (Lyon *et al.* 2014), as telomere length (TL) can be used as a proxy of cellular ageing (Sanders & Newman, 2013). Telomeres cap the ends of DNA, protecting it from damage (Blackburn, 2001). During each cell division the telomere shortens, with an average yearly attrition rate of 14–20 base pairs per year found in cross-sectional studies and a yearly LTL attrition rate of 32–46 base pairs reported in longitudinal studies (Blackburn, 1991; Cawthon *et al.* 2003; Muezzinler *et al.* 2013). When telomeres are at a critically short length, cells become susceptible to senescence and apoptosis (Blackburn, 2001). Moreover, chromosome ends can activate DNA damage-response pathways, in turn this can lead to genetic mutations and altered gene expression, thus leading to susceptibility to various diseases (Lyon *et al.* 2014).

The first studies relating personality to TL showed mixed results. Sadahiro *et al.* found a cross-sectional relation between low conscientiousness and shorter TL, yet unexpectedly reported that higher scores on neuroticism were related to longer TL (Sadahiro *et al.* 2015). In contrast, results of the longitudinal study by van Ockenburg *et al.* showed that persons who score high on neuroticism have shorter TL (Van Ockenburg *et al.* 2014). Two cross-sectional null-studies relating TL to the traditional Big Five in an elderly population (Savolainen *et al.* 2015) and the less traditional Type D personality trait in patients with chronic heart failure (Huzen *et al.* 2010) were also published. In the latter study, the authors also did not find a relation between depression and TL (Huzen *et al.* 2010), whereas previous studies did report a negative association between the two (Verhoeven *et al.* 2014; Schutte & Malouff, 2015). As we know that personality traits (e.g. neuroticism and Type D personality) can be a risk factor for poor mental health, such as the development of depression (Denollet *et al.* 1996; Denollet, 2005; Kendler *et al.* 2006) information on mental health is warranted when relating personality to TL. Discrepancies among these studies relating personality to TL may be inherent to the study design as affirmative studies included younger individuals. These disparities may be intrinsic to whether or not researchers controlled for lifestyle factors as in the study by Sadahiro *et al.* significant associations were found while adjusting for age and gender (Sadahiro *et al.* 2015), whereas Savolainen and colleagues reported non-significant results while adjusting additionally for education, presence of chronic illnesses, depressive symptoms, and the lifestyle factors: BMI, alcohol use, smoking and physical activity (Savolainen *et al.* 2015).

The objective of this 6-year longitudinal study is to examine the relation between personality (i.e. the Big Five and Type D personality traits) and TL measured in leukocytes (LTL) across two time points among adults with a wide age-range, with and without the inclusion of lifestyle factors. In line with the current literature, we hypothesized that lower conscientiousness is related to shorter LTL. Moreover, we speculated that higher neuroticism, lower agreeableness, and Type D personality may be related to shorter LTL, given their associations with adverse health outcomes. Additionally, we examined whether the relation between personality and LTL differed by having a depression and/or anxiety disorder. Finally, we examined whether psychiatric status influenced the relation between personality and LTL.

## Methods

### Participants

Data are from the Netherlands Study of Depression and Anxiety (NESDA), an ongoing longitudinal multi-site cohort study examining predictors, course and consequences of depression and anxiety. A detailed description of NESDA can be found elsewhere (Penninx *et al.* 2008). In short, the NESDA sample consists of 2981 persons between 18 and 65 years old, recruited between September 2004 and February 2007 from community, primary and specialized mental health care settings, including persons with a psychiatric diagnosis (depressive and/or anxiety disorder) and healthy controls. Exclusion criteria were; not being fluent in Dutch, and having a primary clinical diagnosis of another other severe mental disorder (e.g. bipolar disorder, obsessive-compulsive disorder, post-traumatic stress disorder, severe substance use disorder, or a psychotic disorder), which were either self-reported or reported by their mental health practitioner. A detailed description of the number of excluded participants for each site is described elsewhere (Penninx *et al.* 2008). The NESDA study was approved by the local Ethics Committees of each participating center and all participants signed an informed consent form.

At baseline, data collection entailed a medical examination, blood draw, filling-out questionnaires and an interview. Overall, 2936 persons had LTL at baseline and 1883 persons had LTL at 6-year follow-up (FU6). Persons who did not participate at FU6 had longer baseline LTL, were slightly younger, had less educational years, were less physically active, were less often a former smoker, and were more often a non-drinker (all *p*’s < 0.05). They moreover scored slightly higher on neuroticism, yet slightly lower on extraversion, openness to experience, and agreeableness (all *p*’s < 0.01). Groups did not differ with respect to Type D personality, gender, somatic health, body mass index (BMI), and recent life stress (all *p*’s > 0.05).

### Measurements

#### Personality

##### Big Five personality traits

The Big Five personality traits were measured by the revised NEO Five-Factor Inventory (NEO-FFI). This widely used 60-item questionnaire is answered on a 5-point Likert scale (range 1–5) and is designed to measure the five personality traits: neuroticism, extraversion, openness to experience, agreeableness, and conscientiousness (Costa & McCrae, 1995). All five scales show good internal consistency (Costa & Mccrae, 1992). The NEO-FFI was measured at baseline and at 2-year follow up (FU2). At FU4 only the neuroticism and extraversion subscales were re-assessed. To maximize the sample size, an average personality score across time was calculated. That is, mean scores for each personality trait were calculated by dividing the sum of all measurements by the number of available measurements across time. The Big Five personality traits are stable across time (Rantanen *et al.* 2007), which was confirmed in this sample by the strong correlations between scales at all time points (all *r*’s > 0.70, see online Supplementary Table S1). Mean personality traits correlated very strongly with each individual time point (all *r*’s > 0.91, see online Supplementary Table S1).

##### Type D personality

The distressed (Type D) personality, was assessed at FU6 by the Dutch 14-item Type D personality scale (DS14) (Denollet, 2005). Type D personality is the tendency to experience both negative affectivity (NA) and social inhibition (SI) (Denollet, 2005) and is stable over time (Kupper *et al.* 2011). Items are answered on a 5-point Likert scale (range 0–4). In this study we used two methods to assess Type D personality. First, Type D personality was defined as a cut-off score of ⩾10 on both subscales of the DS14, and coded as a dichotomous variable labeling individuals with *v.* without a Type D personality (Denollet, 2005). Second, we analyzed the continuous scores on negative affectivity and social inhibition, as well as their interaction term (NA × SI), which reflects a continuous proxy measure of Type D personality (Smith, 2011; Denollet *et al.* 2013).

#### Leukocyte telomere length

At baseline and FU6 LTL was measured. Fasting blood samples were drawn from participants between 8:30 and 9:30 AM. Peripheral blood mononuclear cells from all samples were isolated from whole blood using density-gradient centrifugation (with Ficoll-Plaque PLUS) and stored in a −20 °C freezer. DNA was extracted with Gentra PureGene (samples collected 2003–2004), Qiagen FlexiGene (2005–2007), Gentra PureGene (2007–2009) and Chemagen (2009–2014). Quality control checks assured us good quality of the extracted DNA. LTL was determined using an adapted quantitative polymerase chain reaction (PCR) (Verhoeven *et al.* 2014). LTL at baseline was determined at the laboratory of Telomere Diagnostics, Inc (Menlo Park, CA) and at FU6 at the University of California, San Francisco. Each patients’ sample (T) was run in triplicate wells in the 384-well assay plate and compared with a single gene copy number (S), relative to a reference sample to generate a T/S ratio proportional to mean LTL (Cawthon, 2002; Aviv *et al.* 2011). A detailed description of this method is described elsewhere (Verhoeven *et al.* 2014). The T/S ratio was converted to base pairs (bp) using this formula: bp = 3274 + 2413 × ((*T*/*S* − 0.0545)/1.16).

Reliability of both assays was adequate: the inter-assay coefficient variation (CV) (baseline: CV = 4.6%; 6-year: CV = 3.0%) was sufficiently low as shown by the included quality control DNA samples on each PCR run. Similar conclusions can be drawn based on the telomere (baseline: CV = 2.0%; FU6: CV = 5.4%) and the single-gene assay (baseline: CV = 1.6%; FU6: CV = 4.8%) separately. There were systematic differences between baseline and FU6 LTL measurements, as different reference samples were used. Therefore, T/S ratios at FU6 were adjusted relative to the baseline samples, by rerunning and comparing baseline sample plates (*n* = 226, up to eight samples from each of the baseline plates) to FU6. On average, the T/S ratios of the FU6 runs were at 76% of the T/S ratios of baseline, hence the follow-up T/S ratios were divided by 0.76 to correct for systematic differences. Importantly, DNA samples were de-identified, the laboratories that performed the assays were blind to all other measurements, and case and control samples were randomly distributed over plates.

#### Covariates

Covariates were assessed at both baseline and FU6 through self-reports via questionnaires or during the interview. Education was assessed in years of education during the interview. The number of medical conditions (i.e. diabetes, osteoarthritis, stroke, cancer, heart, chronic lung, intestinal or thyroid diseases) for which a person was receiving medical treatment was counted during the interview, and was a proxy for somatic health. Furthermore, lifestyle factors were assessed. BMI was calculated by dividing a persons’ weight by their squared height measured at medical examination. Alcohol consumption based on questionnaire data was grouped based on the number of drinks per week, into non-drinker, mild-moderate (<14 and <21 drinks per week for women and men, respectively) and severe drinker (⩾14 and ⩾21 drinks per week for women and men, respectively). Smoking status assessed by a questionnaire was categorized into never smoked, former smoker, and current smoker. Former smokers at baseline were categorized if they indicated to have ever smoked but are currently not smoking. At follow-up they were categorized as former smoker if they answered smoker on a previous measurement but at the current follow-up measurement report that they currently do not smoke. Physical activity as assessed by the International Physical Activity questionnaire (IPAQ) (Craig *et al.* 2003) was expressed as overall energy expenditure in Metabolic Equivalent Total (MET) minutes per week. Recent life stress, defined as the count of 12 negative life events during the past year as assessed during the interview by using the Brugha questionnaire (Brugha & Cragg, 1990) was considered, as this has shown to be related to LTL (Van Ockenburg *et al.* 2015; Verhoeven *et al.* 2015*b*). Finally, depression and anxiety disorders were assessed at baseline and FU6 by the DSM-IV Composite International Diagnostic Interview version 2.1 and included major depressive disorder, dysthymia, panic disorders, social phobia, agoraphobia, and generalized anxiety disorder. In the NESDA study, psychiatric disorder status (depressed and/or anxious) has been negatively associated with LTL (Verhoeven *et al.* 2014; Verhoeven *et al.* 2015*a*). To see whether any associations between personality and LTL differed by psychiatric disorder status, we categorized persons as having a current (6-month period) psychiatric diagnosis (depressed and/or anxious). Additionally, because psychiatric status is related to LTL in this patient sample (Verhoeven *et al.* 2014), and personality (e.g. neuroticism) can be seen as a risk factor for developing psychiatric conditions (Denollet *et al.* 1996; Denollet, 2005; Kendler *et al.* 2006), psychiatric status could influence the relation between personality and LTL. We therefore included psychiatric status as a covariate in statistical analyses.

### Statistical analyses

Sample characteristics for those included at baseline (*n* = 2936) and FU6 (*n* = 1883) are presented as means (standard deviation, s.d.) or numbers (percentages). The relations between sociodemographics, somatic health, lifestyle factors, and recent life stress with LTL at both time points were tested by means of generalized estimated equations (GEE) analyses.

To examine whether personality was consistently related to LTL, GEE analyses were conducted with personality as predictors (mean scores for each of the Big Five personality traits and Type D information at FU4) and LTL at both time points as outcome variable. To take within-person correlations as a result of multiple observations per person into account, GEE analyses were performed with exchangeable correlation structure and identity log-link function (Twisk, 2004). Time, that is the within subject variable defining the order of measurements, was categorized as 1 (baseline) and 2 (FU6). Separate analyses were performed for each of the Big Five personality traits and Type D personality.

Analyses included covariates measured at both time points, which were entered in two steps. First, we adjusted for sociodemographic factors (age, gender and education), which were seen as potential confounders. Secondly, somatic health, lifestyle factors (smoking alcohol use, BMI and physical activity), and recent life stress at both time points were considered as possibly explanatory variables and added to the model. In order to examine whether effects were consistent across time, age, and gender, interaction terms between personality trait (mean Big Five traits/Type D at FU4)×time/age/gender together with their standardized main effects were added to the sociodemographic adjusted models. Adding time interactions allowed us to examine the association between personality and LTL attrition. No interaction terms for the continuous Type D personality scoring was calculated, as a third way interaction (NA × SI × time/age/gender/psychiatric status) is less comprehensible.

Because short LTL is associated with psychiatric disorders such as depression and anxiety disorders (Verhoeven *et al.* 2014; Verhoeven *et al.* 2015*a*), we examined whether the relation between personality and LTL differed by depression and/or anxiety disorder status by adding the interaction term personality × psychiatric disorder. Furthermore, we examined whether psychiatric status influenced the relation between personality and LTL, by adding psychiatric status as a covariate to the model.

Because there was attrition from baseline to follow-up due to drop-out of 36%, which was not completely at random, analyses were performed using multiple imputation obtaining 40 imputed datasets. Details on the imputation method are provided in the online Supplementary Material File. Pooled estimates were used to obtain statistical interference. Additionally, we performed two types of sensitivity analyses: (1) using the original non-imputed data; and (2) including only the 64% individuals who had data available at both measurement points.

All analyses were conducted in SPSS version 22. Statistical significance was set at *p* < 0.05, evaluation of trends was set at *p* < 0.10. We chose not to use a more stringent alpha level since this is the first study relating both the Big Five personality traits and Type D personality to LTL in both a healthy sample and psychiatric patients, hence we wanted to avoid making a type-2 error.

## Results

### Study population

Table 1 shows characteristics of the study population at baseline (*n* = 2936) and FU6 (*n* = 1883). Participants were on average 41.8 years old at baseline and two-third was women. Most participants were current or former smokers and modest drinkers. The average BMI was approximately 25, whereas little more than half of participants had a current psychiatric disorder. The number of negative life events during the past year was 0.2 at baseline and 1.3 at FU6. Mean scores on the Big Five personality traits ranged from 35 for neuroticism to 44 on agreeableness. Nearly 32% of participants were classified as having a Type D personality (Table 2).

**Table 1.** Participant characteristics at baseline and FU6

	Baseline (*n* = 2936)	FU6 (*n* = 1883)
Sociodemographics		
Age, mean years (s.d.)	41.8 (13.1)	48.6 (12.9)
Gender (female), N (%)	1950 (66.4)	1232 (65.4)
Mean years of education (s.d.)	12.2 (3.3)	12.9 (3.3)
Somatic health (number of somatic diseases), M (s.d.)	0.6 (0.9)	0.6 (0.9)
Lifestyle		
Smoking, N (%)		
Never	826 (28.1)	555 (29.5)
Former	974 (33.2)	796 (42.3)
Current	1136 (38.7)	529 (28.1)
Alcohol use, N (%)		
Nondrinker	500 (17.0)	311 (17.5)
Modest drinker	2063 (70.3)	1294 (72.7)
Heavy drinker	373 (12.7)	176 (9.9)
BMI, M (s.d.)	25.6 (5.1)	24.8 (7.8)
Physical activity in 1000 MET-min/wk, M (s.d.)	3.7 (3.1)	4.0 (3.4)
Recent life stress		
Number of life events in the past year, M (s.d.)	0.2 (0.5)	1.3 (1.2)
Psychiatric status		
Current or remitted depression and/or anxiety disorder, N (%)	884 (46.9)	1355 (72.0)
Leukocyte telomere length		
Base pairs, M (s.d.)	5468 (617)	5387 (433)

N, number; %, percentage; M, mean; s.d., standard deviation; FU6, 6-year follow-up; BMI, body mass index; MET, metabolic equivalent total; min/wk, minutes per week.

**Table 2.** Mean scores on personality traits

Personality traits	Mean (s.d.)
Big Five personality traits, M (s.d.)	
Neuroticism	34.8 (8.5)
Extraversion	37.4 (6.9)
Openness to experience	37.5 (5.4)
Agreeableness	44.0 (5.0)
Conscientiousness	41.9 (6.0)
Type D personality	
Dichotomized (having a Type D personality), N (%)	667 (31.5)
Continuous, M (s.d.)	
Negative affectivity	9.3 (6.6)
Social inhibition	9.6 (6.8)
Type D personality (NA×SI)	113.2 (127.9)

NA×SI, the standardized interaction term between negative affectivity (NA) and social inhibition (SI), representing Type D personality; s.d., standard deviation; N, number; %, percentage.

LTL was 5468 (s.d. = 617) at baseline and 5387 (s.d. = 433) at FU6. GEE analyses relating covariates to LTL at both time points showed that age was negatively related to LTL (*B* = −12.99, *p* < 0.01). Moreover, results showed that females had longer LTL (*B* = 60.98, *p* < 0.01), whereas current smokers had shorter LTL (*B* = −76.57, *p* < 0.01). The other covariates – education, alcohol use, somatic health, BMI, physical activity, and recent life stress – were not significantly related to TL (*p*’s > 0.10, data not shown).

### LTL and personality

Table 3 shows the results of the GEE analyses, based on the imputed data, relating personality to LTL while adjusting for (1) sociodemographics, and (2) somatic health, lifestyle factors, and recent life stress. For the Big Five personality traits, we found a significant relation for neuroticism (*B* = −2.11, *p* = 0.03) and a positive relation for agreeableness (*B* = 3.84, *p* = 0.03) with LTL, after adjustment for sociodemographics. The negative relation for both neuroticism (*B* = −1.99, *p* = 0.06) and agreeableness (*B* = 3.01, *p* = 0.10) with LTL became non-significant, yet there was still a trend after adding the covariates somatic health (i.e. the number of treated medical conditions), lifestyle variables (i.e. smoking, alcohol use, BMI and physical activity), and recent life stress (i.e. the number of negative life events in the past year). There was a statistically significant sociodemographic-adjusted negative relation between Type D and LTL (*B* = −50.16, *p* < 0.01), which did not change after adjusting for somatic health, lifestyle factors, and recent life stress (*B* = −47.37, *p* = 0.01). Analyses relating Type D to LTL by including the continuous scores showed similar results: Type D (NA × SI) was negatively related to LTL (sociodemographic adjustment: *B* = −20.22, *p* = 0.04, full adjustment: *B* = −20.23, *p* = 0.04). Both sensitivity analyses showed similar regression coefficients (see online Supplementary Table S2); yet, in the analyses including only individuals with data at both measurements, some associations became non-significant, which is likely due to reduced power.

**Table 3.** The relation between personality and leukocyte telomere length across two time points

	Leukocyte telomere length (sociodemographic adjustment)			Leukocyte telomere length (full adjustment)		
	*B*	s.e.	*p*	*B*	s.e.	*p*
Big Five personality traits						
Neuroticism	**−2.11**	0.97	0.03*	**−1.99**	1.04	0.06†
Extraversion	0.99	1.22	0.42	0.93	1.32	0.48
Openness to experience	2.08	1.62	0.20	2.68	1.74	0.12
Agreeableness	**3.84**	1.76	0.03*	**3.01**	1.84	0.10
Conscientiousness	1.46	1.34	0.28	1.00	1.41	0.48
Type D personality						
Dichotomized^‡^	**−50.16**	17.82	<0.01*	**−47.37**	18.78	0.01*
Continuous						
Negative affectivity	−16.89	10.94	0.12	−14.29	11.45	0.21
Social inhibition	6.63	11.89	0.58	5.95	12.15	0.63
Type D personality (NA × SI)	**−20.22**	9.81	0.04*	**−20.23**	10.02	0.04*

Note: Results are based on the imputed dataset. Sociodemographic adjusted models are presented: adjusted for age at baseline, gender, and years of education at both time points. Full adjustment: adjusted for sociodemographics and somatic health, lifestyle factors (smoking, alcohol use, BMI, and physical activity), and recent life stress measured at both time points. ^‡^, reference is non Type D personality; NA×SI, the standardized interaction term between negative affectivity (NA) and social inhibition (SI) representing Type D personality. Both standardized main effects (NA and SI) and its interaction term representing Type D personality were entered simultaneously to the model. *p < 0.05 and †*p* < 0.10. *B*-values for significant results and trends are noted in bold.

In order to examine whether associations were consistent across time or not, and hence whether personality was associated with LTL attrition, we added personality × time interaction terms to the sociodemographic models. These analyses showed no statistically significant interactions (Table 4). Both sensitivity analyses showed a significant time interaction effect for Type D (both measurements, *B* = 58.05, *p* = 0.04; and non-imputed data, *B* = 61.16, *p* = 0.02), where persons with Type D personality showed a slower LTL shortening than those without Type D (Table 4). Analyses using the non-imputed data showed an additional significant time interaction for openness to experience (Table 4). Differences in LTL between those scoring high on openness to experience had a faster shortening of LTL than those scoring low on openness to experience (*B* = −26.59, *p* = 0.02).

**Table 4.** The relation between personality and leukocyte telomere length attrition

				Sensitivity analyses					
	Imputed data			Both measurements			Non-imputed data		
	*B*	s.e.	*p*	*B*	s.e.	*p*	*B*	s.e.	*p*
Big Five personality traits									
Neuroticism	**−2.39**	1.28	0.06†	**−2.59**	1.59	0.10	**−2.45**	1.28	0.06†
Neuroticism × time	4.75	13.73	0.73	9.44	12.90	0.46	7.55	11.59	0.52
Extraversion	1.82	1.60	0.26	2.47	1.95	0.20	1.77	1.61	0.27
Extraversion × time	−11.55	13.52	0.39	−15.86	12.71	0.21	−15.56	11.50	0.18
Openness to experience	**3.85**	2.12	0.07†	**2.40**	2.67	0.67	**4.03**	2.13	0.06†
Openness to experience × time	−19.03	13.35	0.15	−22.99	13.25	0.08†	−26.59	11.76	0.02*
Agreeableness	**5.33**	2.20	0.02*	**5.84**	2.83	0.04*	**5.42**	2.21	0.01*
Agreeableness × time	−15.01	14.21	0.29	−22.17	13.64	0.10	**−21.33**	12.13	0.08†
Conscientiousness	1.75	1.78	0.33	2.20	2.15	0.31	1.72	1.79	0.34
Conscientiousness × time	−3.43	13.36	0.80	−5.52	12.06	0.65	−4.38	10.95	0.69
Type D personality									
Dichotomized^‡^	**−66.96**	23.64	<0.01*	**−77.17**	29.47	<0.01*	**−80.58**	27.36	<0.01*
Dichotomized × time	33.58	28.43	0.24	58.05	27.99	0.04*	61.16	26.82	0.02*

Note: Sociodemographic adjustment: adjusted for age at baseline, gender, and years of education at both time points. ^‡^, reference is non Type D personality. The number of observations in the non-imputed dataset differed for each analysis ranging from *n* = 3766 (1883 + 1883) when relating Type D personality to LTL, up to *n* = 4819 (2936 + 1883) when relating the Big Five personality traits to LTL. No time interaction for the continuous Type D personality scoring was calculated, as a third way interaction (NA × SI × time) is less comprehensible. *p < 0.05; †*p* < 0.10* *B*-values for significant results and trends in the main (imputed) analyses are denoted in bold for all three datasets.

There were no age and gender moderation effects for LTL (*p*’s > 0.10, data not shown). Furthermore, we examined whether the relation between personality and LTL was different by psychiatric status, that is, those with a current depression and/or anxiety disorder *v.* those without. No significant interaction effects were found for psychiatric status for the relation between personality and baseline LTL (*p*’*s* > 0.10, data not shown). Hence, the relation between personality and baseline LTL was similar among those with and without a current depression and/or anxiety disorder. Both types of sensitivity analyses provided similar results (data not shown). Entering psychiatric status as a covariate showed similar associations between LTL and neuroticism (*B* = −1.69, *p* = 0.12); and agreeableness (*B* = 3.44, *p* = 0.05), yet relations became non-significant. Type D personality remained significantly associated with LTL after adjustment for psychiatric status (dichotomized: *B* = −45.86, *p* = 0.02; and NA × SI: *B* = −20.14, *p* = 0.04).

## Discussion

This longitudinal study related the well-established Big Five personality traits and the more recently developed Type D personality construct to LTL measured across two time points. Results of this study confirmed our expectations that high neuroticism and low agreeableness were related to shorter LTL measured at two time points. However, these associations became statistically non-significant after adjusting for somatic health, lifestyle factors, and recent life stress. Importantly, Type D personality was negatively related to LTL measured at two time points, which remained significant after full adjustment. Personality was not associated with LTL attrition across time in our main analyses. Associations between personality and LTL did not differ by age, gender, and current psychiatric status. Adding psychiatric status to the model, resulted in non-significant associations for neuroticism and agreeableness, whereas Type D personality remained significantly related to LTL.

Contrary to our hypothesis, we did not find an association between conscientiousness and LTL. This lack of association between conscientiousness and LTL was unexpected, as previous research has consistently reported that higher conscientiousness is related to decreased mortality (Kern & Friedman, 2008; Jokela *et al.* 2013) and more recently to LTL (Sadahiro *et al.* 2015). Dissimilarities between the significant association with LTL found by Sadahiro *et al.* and our study may be allocated to differences in age; they included a group of Japanese students with a mean age of 23 (s.d. = 1.7) (Sadahiro *et al.* 2015), whereas we included an older sample with a broad age range (mean age = 42, s.d. = 13.1). This could be the result of different attrition rates across the life span as research has shown that early in life one has a fast LTL attrition rate, whereas during adulthood attrition is considered relatively small (Hjelmborg *et al.* 2015). The relation between lower agreeableness and shorter LTL, is in agreement with previous findings of Savolainen and colleagues who found a similar relation among women but not in men in an elderly population (Savolainen *et al.* 2015). Our findings underscore the previously described protective character of higher agreeableness with respect to mortality (Weiss & Costa, 2005). Contrary to the null finding in the study by Sadahiro *et al.* (2015) we confirmed the relation found by others (Van Ockenburg *et al.* 2014) relating higher neuroticism to shorter LTL among adults of a broad age-range. Even after full adjustment there was still a trend. Some associations between the Big Five personality traits and LTL became non-significant after adjustment for covariates but similar trends still remained. Hence, the relations are influenced but not completely driven by differences in somatic health, lifestyle or experienced negative life events. Moreover, our study showed that persons with a Type D personality had shorter LTL measured at both time points, even after full adjustment.

Although there were no significant time interactions in the main analyses, the analyses based on the non-imputed data showed a significant time interaction for openness to experience and Type D personality, which are somewhat unexpected based on the accelerated aging hypothesis. These time interactions were not confirmed in our main analyses nor in the sensitivity analyses using individuals with complete LTL assessments, therefore it is unclear whether we found a true effect in the non-imputed analyses.

Important in the interpretation of our results is the crucial question as to how Type D personality is understood in terms of the renowned Big Five personality traits. The Type D personality sub-traits negative affectivity and social inhibition are known to correlate most strongly with neuroticism and extraversion (reversed) of the Big Five taxonomy (De Fruyt & Denollet, 2002; Horwood *et al.* 2015), which was confirmed in our study. Previous studies have estimated that neuroticism and extraversion, although related to Type D personality, are not similar to Type D personality, given that both traits together explained half of the variance of the Type D construct (De Fruyt & Denollet, 2002). A second important issue is the relation between neuroticism and Type D personality with depression as the NESDA sample consists of persons with a current depression and/or anxiety disorder and healthy controls. Although some overlap exists regarding neuroticism, the Type D sub-trait negative affectivity, and depression, the conceptual difference lies within the fact that personality is a permanent trait while depression is a disorder and thus a more temporary state (Denollet *et al.* 2009; Karsten *et al.* 2012). Psychometrically, depression loads on different higher-order constructs or factors than Type D personality and its sub-traits negative affectivity and social inhibition (Pelle *et al.* 2009). Underscoring the distinctiveness between depression and the personality trait neuroticism and Type D personality, we found that the relations between neuroticism and Type D with LTL were similar among those with and without a current depressive and/or anxiety disorder. Results of the analyses where we added current psychiatric status as a covariant to the model – examining whether psychiatric status influences the relation between personality and LTL – showed similar but non-significant associations for neuroticism and agreeableness with LTL. Contrary, the relation between Type D personality and LTL was unchanged after adding current psychiatric status. These findings suggest that current psychiatric status – that is, being currently diagnosed with a depression and/or anxiety disorder – could be a potential confounder or may be a possible mechanism or pathway for the relation between neuroticism and agreeableness with LTL, but not so much for Type D personality.

Neuroticism, agreeableness, Type D personality and shorter LTL are associated with morbidity, such as the age-related diseases cancer and CVD, and mortality (Wilson *et al.* 2004; Denollet, 2005; Weiss & Costa, 2005; Chapman *et al.* 2010; Mols *et al.* 2012; Denollet, 2013; Denollet *et al.* 2013; Lyon *et al.* 2014; McCann, 2014). Similar underlying risk factors, such as the amount of oxidative stress exposure, inflammation and a genetic vulnerability may be responsible for these associations. Experiencing psychological stress leads to the release of the stress hormone cortisol, which increases damage by oxidative stress (McIntosh *et al.* 1998) and inflammation (Epel, 2009). Neuroticism and Type D personality are associated with experiencing psychological stress (Denollet *et al.* 1996; Denollet, 2005; Kendler *et al.* 2006), which is accompanied by increased oxidative stress and inflammation. Hence, elevated stress hormones may be a mediating factor partly explaining the relations between personality (i.e. neuroticism, agreeableness, and Type D personality) and LTL, mortality and morbidity. In clinical practice, the primary focus should not be on changing personality itself, but rather on providing these individuals with the skills necessary to more effectively cope with stressful events.

A major strength of this study is its large sample size and longitudinal design. Moreover, our study included well characterized patients with a current depressive and/or anxious disorder together with healthy controls. Also, our sample comprised a wide age range, and included information on important covariates such as somatic health, lifestyle factors and recent life stress. Furthermore, LTL was reliably measured with qPCR, whereas the intra-assay coefficients of variation were sufficiently low. As a result we could comprehensively examine the relation between personality and LTL measured across a 6-year period.

It is however also worth mentioning the limitations of this study. First, longitudinal data was analyzed where Type D personality was measured 6 years after baseline LTL was assessed, whereas the Big Five personality traits were measured at several occasions and scores were averaged. The Big Five and Type D personality are considered to be stable across time (Rantanen *et al.* 2007; Kupper *et al.* 2011), which is supported by the high correlations between mean Big Five personality traits and scores on each measurement occasion. We therefore believe that averaging personality scores across time had little impact on our results. Furthermore, no conclusions regarding causality can be drawn. Following from this, we have assumed that personality may affect LTL, yet research has also shown that LTL deletion can cause neuropsychological abnormalities (Zhang *et al.* 2007). By analogy, short LTL might also have an impact on personality through increasing levels of inflammation (Conraads *et al.* 2006; Sutin *et al.* 2010). Additionally, the difference in the effects between Type D personality as a dichotomized measure and the continuous Big Five traits may be an artifact of the nature of the variable rather than a true difference. Although the small differences in results for the imputed analyses compared with both sensitivity analyses suggest that missing data had limited impact on the observed findings, as LTL at both measurements was not missing at random. There seems to be an impact of missingness of LTL when examining whether personality is associated with LTL attrition, given the discrepancy in results from the imputed *v.* sensitivity analyses. Furthermore, as is common in studies examining TL, LTL was used as a valid and often used indicator of cellular ageing. Nonetheless, average TL consists of several cell types complicating the distinguishing between TL differences due to actual shortening or lengthening or the reorganization of cell types (Lin *et al.* 2015). Additionally, no information on telomerase activity was available in our study, inhibiting the studying of its important role in maintaining TL. Also, LTL from baseline and FU6 was measured in two different batches two years apart which could have introduced noise between time-points. To adjust for possible systematic differences, samples from both time points were re-rerun together and FU TLT was converted accordingly.

In conclusion, this study demonstrates that high neuroticism and low agreeableness were associated with shorter LTL measured across a 6-year period. After controlling for somatic health, lifestyle factors, and recent life stress (full adjustment) associations between these Big Five traits became non-significant, although similar trends were still observed. However, the association between Type D personality and shorter LTL measured across a 6-year period remained significant, even after full adjustment. It is important to note, that the relationship between personality and LTL did not differ by age, gender or a current depressive and/or anxiety disorder. Future research should explore causality as personality may lead to a predisposed vulnerability leading to biological ageing.

## Supplementary material

For supplementary material accompanying this paper visit http://dx.doi.org/10.1017/S0033291717002471. click here to view supplementary material
