# Supplementary material for: Leukocyte telomere length and personality: associations with the Big Five and Type D personality traits
Source: Psychol Med. 2017 Sep 11;48(6):1008–19. doi: 10.1017/S0033291717002471 (PMC5851042; doi:10.1017/S0033291717002471)
Supplement: Supplementary file 1 [file S0033291717002471sup001.docx]

Table S1: Correlations between the Big Five personality traits and Type D personality

|  | Big Five traits (baseline) | | | | | Big Five traits (FU2) | | | | | Big Five traits (FU4) | | Type D (FU6) | | | |
| --- | --- | --- | --- | --- | --- | --- | --- | --- | --- | --- | --- | --- | --- | --- | --- | --- |
|  | Neuro | Extrav | Open | Agreea | Consc | Neuro | Extrav | Open | Agreea | Consc | Neuro | Extrav | Yes/no | NA | SI | NA*SI |
| Big Five traits (baseline) |  |  |  |  |  |  |  |  |  |  |  |  |  |  |  |  |
| Neuroticisme | 1 | -.598 | -.033 | -.314 | -.511 |  |  |  |  |  |  |  |  |  |  |  |
| Extraversion | -- | 1 | .101 | .302 | .485 |  |  |  |  |  |  |  |  |  |  |  |
| Openness to experience | -- |  | 1 | .109 | -.034 |  |  |  |  |  |  |  |  |  |  |  |
| Agreeableness | -- | -- | -- | 1 | .253 |  |  |  |  |  |  |  |  |  |  |  |
| Conscientiousness | -- | -- | -- | -- | 1 |  |  |  |  |  |  |  |  |  |  |  |
|  |  |  |  |  |  |  |  |  |  |  |  |  |  |  |  |  |
| Big Five traits (FU2) |  |  |  |  |  |  |  |  |  |  |  |  |  |  |  |  |
| Neuroticism | .775 | -.487 | -.028 | -.244 | -.437 | 1 | -.593 | -.032 | -.309 | -.539 |  |  |  |  |  |  |
| Extraversion | -.527 | .798 | .064 | .280 | .443 | -- | 1 | .079 | .311 | .502 |  |  |  |  |  |  |
| Openness to experience | .000 | .049 | .749 | .117 | -.039 | -- | -- | 1 | .078 | -.030 |  |  |  |  |  |  |
| Agreeableness | -.254 | .252 | .058 | .708 | .208 | -- | -- | -- | 1 | .265 |  |  |  |  |  |  |
| Conscientiousness | -.449 | .386 | -.086 | .181 | .743 |  |  |  |  | 1 |  |  |  |  |  |  |
|  |  |  |  |  |  |  |  |  |  |  |  |  |  |  |  |  |
| Big Five traits (FU4) |  |  |  |  |  |  |  |  |  |  |  |  |  |  |  |  |
| Neuroticisme | .738 | -.520 | .002 | -.210 | -.424 | .797 | -.524 | -.025 | -.225 | -.474 | 1 | -.602 |  |  |  |  |
| Extraversion | -.494 | .768 | .062 | .241 | .376 | -.544 | .824 | .277 | .449 | -.611 | -- | 1 |  |  |  |  |
|  |  |  |  |  |  |  |  |  |  |  |  |  |  |  |  |  |
| Type D (FU6) |  |  |  |  |  |  |  |  |  |  |  |  |  |  |  |  |
| Dichotomized (yes/no) | .463 | -.447 | -.036 | -.250 | -.315 | .500 | -.447 | -.030 | -.256 | -.352 | .533 | -.505 | 1 | .676 | .685 | .789 |
| Continuous |  |  |  |  |  |  |  |  |  |  |  |  |  |  |  |  |
| NA | .625 | -.478 | -.022 | -.304 | -.361 | .671 | -.515 | -.030 | -.325 | -.422 | .692 | -.524 | -- | 1 | .539 | .135 |
| SI | .436 | -.586 | -.056 | -.250 | -.334 | .462 | -.604 | -.045 | -.280 | -.359 | .492 | -.634 | -- | -- | 1 | .213 |
| NA*SI | .036 | -.075 | -.056 | -.070 | -.035 | .074 | -.080 | -.050 | -.049 | -.045 | .089 | -.106 | -- | -- | -- | 1 |
|  |  |  |  |  |  |  |  |  |  |  |  |  |  |  |  |  |
| Big Five traits mean scores |  |  |  |  |  |  |  |  |  |  |  |  |  |  |  |  |
| Neuroticism | .927 | -.596 | -.036 | -.295 | -.504 | .932 | -.579 | -.032 | -.286 | -.521 | .914 | -.595 | .539 | .714 | .498 | .074 |
| Extraversion | -.574 | .934 | .093 | .293 | .456 | -.596 | .940 | .077 | .312 | .501 | -.594 | .930 | -.511 | -.541 | -.651 | -.100 |
| Openness to experience | -.040 | .097 | .950 | .092 | -.056 | -.017 | .069 | .928 | .105 | -.036 | -.011 | .073 | -.035 | -.027 | -053 | -.057 |
| Agreeableness | -.302 | .311 | .124 | .939 | .240 | -.304 | .304 | .074 | .925 | .255 | -.229 | .275 | -.274 | -.333 | -.281 | -.061 |
| Conscientiousness | -.510 | .496 | -.031 | .252 | .946 | -.528 | .474 | -.062 | .237 | .931 | -.478 | .439 | -.356 | -.414 | -.370 | -.045 |

Note: Neuro=neuroticism; Extrav=extraversion; Open=openness to experience; Agreea=agreeableness; Consc=conscientiousness; FU2= two year follow-up; FU4=four year follow-up; FU6= six year follow-up; NA=negative affectivity; SI=social inhibition; NA*SI=the standardized interaction term between negative affectivity (NA) and social inhibition (SI), this interaction term represents the Type D personality.

|  | **Imputed data** | | |  | **Sensitivity analyses** | | | | | | | | |  |  |
| --- | --- | --- | --- | --- | --- | --- | --- | --- | --- | --- | --- | --- | --- | --- | --- |
|  |  | | |  | Both measurements | | |  | Not imputed data | | | | |  |  |
|  | B | SE | P |  | B | SE | P |  | B | SE | | P | |  |  |
| **Big Five personality traits** | | | | | | | |  |  | | | | |  |  |
| Neuroticism | **-2.11** | 0.97 | 0.03* |  | **-2.05** | 1.17 | 0.08† |  | **-2.15** | | 1.05 | | 0.04* |  |  |
| Extraversion | 0.99 | 1.22 | 0.42 |  | 1.33 | 1.43 | 0.35 |  | 0.96 | | 1.30 | | 0.46 |  |  |
| Openness to experience | 2.08 | 1.62 | 0.20 |  | 0.28 | 1.94 | 0.89 |  | 2.31 | | 1.74 | | 0.18 |  |  |
| Agreeableness | **3.84** | 1.76 | 0.03* |  | **3.64** | 2.10 | 0.08† |  | **3.98** | | 1.83 | | 0.03* |  |  |
| Conscientiousness | 1.46 | 1.34 | 0.28 |  | 1.73 | 1.55 | 0.26 |  | 1.45 | | 1.42 | | 0.31 |  |  |
|  |  |  |  |  |  |  |  |  |  | | | | |  |  |
| **Type D personality** | | | | | | | |  |  | | | | |  |  |
| Dichotomized^ⱡ^ | **-50.16** | 17.82 | <0.01* |  | **-48.14** | 21.15 | 0.02* |  | **-53.02** | | 20.38 | | 0.01* |  |  |
| Continuous |  |  |  |  |  |  |  |  |  | | | | |  |  |
| NA | -16.89 | 10.94 | 0.12 |  | -15.66 | 12.17 | 0.20 |  | -16.31 | | 12.19 | | 0.18 |  |  |
| SI | 6.63 | 11.89 | 0.58 |  | 4.11 | 11.98 | 0.73 |  | 6.37 | | 11.80 | | 0.59 |  |  |
| NA x SI (Type D) | **-20.22** | 9.81 | 0.04* |  | **-16.38** | 10.12 | 0.11 |  | **-19.26** | | 10.00 | | 0.05† |  |  |

Table S2: Original and sensitivity analyses relating personality to LTL over two time points

Note: Sociodemographic adjusted models are presented: adjusted for age at baseline, gender, and years of education at both time points. ^ⱡ^ = reference is non Type D personality; NA=negative affectivity; SI=social inhibition; NA*SI=the standardized interaction term between negative affectivity (NA) and social inhibition (SI). NA, SI and its interaction term (NA*SI) are entered simultaneously to the model, the interaction term representing the Type D personality. Standardized coefficients that were significant in the imputed analyses are denoted in bold for all three analyses. * p<0.05; † p<0.10

**Multiple Imputation method used**

Recent literature describes various imputation techniques such as ‘just another variable’ (JAV) based on first computing the transformation (i.e. computation of the interaction effect) and then including the transformation (i.e. interaction) in the imputation model.[1] Although these methods are aimed at reducing bias in the imputed interaction terms, it actually introduces bias when used on missing data which are not completely at random (MCAR)[2-4] or in other model types than linear regression analyses[2, 4]. Given that our missing data is MCAR (that is in fact the reason why we need to imputate data, if data was MCAR we could use GEE as this is one of its assumptions) and we use GEE analyses, imputation methods such as JAV where transformation before imputation takes place is not (yet) plausible. The multiple imputation (MI) model using the automatic function in SPSS – that is, the automatic determination of the order of imputation based on least to most missing values for each variable to detect a monotone pattern of missingness – was used. Based on our dataset a non-monotone pattern was detected, hence the Fully Conditional Specification (FCS) was used. All variables used in the GEE analyses were included in the imputation model. As our missingness was not MCAR, the interaction terms for personality and age/sex/psychiatric status/time were not included in the imputation model. The standardized main effects used to calculate the interaction terms after imputation were included in the imputation model. Type D personality (standardized negative affectivity * standardized social inhibition) as an interaction term was included in the imputation model as such. The final order of variables is presented below. Forty imputed datasets were obtained and pooled estimates were calculated to obtain statistical interference.

Order of imputed variables
Gender; (standardized) age; time; (standardized) neuroticism, extraversion, agreeableness, consciousness, openness to experience; somatic health; education; BMI; psychiatric status; recent life stress; smoking status; alcohol status; physical status; LTL; standardized Social inhibition (Type D trait); dichotomized Type D variable; standardized Negative affectivity (Type D trait); standardized interaction term Negative affectivity * Social Inhibition (Type D)

*[1] Von Hippel PT: How to impute interactions, squares and other transformed variables. Sociological Methodology. 2009, 39: 265-291. DOI: 10.1111/j.1467-9531.2009.01215.x.*

# *[2] Seaman SR, Barlett JW, and White IR. Multiple imputation of missing covariates with non-linear effects and interactions: an evaluation of statistical methods. BMC Medical Research Methodology. 2012. DOI: 10.1186/1471-2288-12-46*

# *[3] Morris TP, White IR, Royston P. Tuning multiple imputation by predictive mean matching and local residual draws. 2014. DOI: 10.1186/1471-2288-14-75*

*[4] Barlett JW, Seaman SR, White IR, Carpenter JR, for Alzheimer’s Disease Neuroimaging Initiative. 2015, 24: 462-487. DOI:* [*10.1177/0962280214521348*](https://doi.org/10.1177/0962280214521348)
